# Reconstruction of maxillofacial bone defects using patient-specific long-lasting titanium implants

**DOI:** 10.1038/s41598-022-11200-0

**Published:** 2022-05-09

**Authors:** Ho-Kyung Lim, Young-Jun Choi, Won-Cheul Choi, In-Seok Song, Ui-Lyong Lee

**Affiliations:** 1 Department of Oral and Maxillofacial Surgery, Korea University Guro Hospital, Seoul, Republic of Korea; 2 Department of Oral & Maxillofacial Surgery, Chung-Ang University College of Medicine, Seoul, Republic of Korea; 3 Department of Orthodontics, Dental Center, Chung-Ang University Hospital, Seoul, Republic of Korea; 4 Department of Oral & Maxillofacial Surgery, Korea University Anam Hospital, Seoul, Republic of Korea; 5 Department of Oral & Maxillofacial Surgery, Dental Center, Chung-Ang University Hospital, Seoul, Republic of Korea

**Keywords:** Dentistry, Prognosis, Therapeutics, Oral cancer, Dental diseases, Oral diseases, Trauma

## Abstract

The objective of this retrospective study is to verify the effectiveness and safety of patient-specific titanium implants on maxillofacial bones, with a long-term follow-up. Total 16 patients with various maxillofacial defects underwent reconstruction using patient-specific titanium implants. Titanium implants, manufactured by electron beam melting, selective laser sintering, or milling, were inserted into the maxilla, mandible, or zygoma. Long-term follow‐up (36.7 ± 20.1 months) was conducted after the surgery. Bone fusion of the titanium implant body, postoperative infection, implant malunion, functional results, patient satisfaction, subsidence, osteolysis around the implants, and complications were recorded and analyzed at the last follow-up. Of the 28 implants, only one failed to unite with the bone; therefore, revision surgery was performed. No osteolysis or subsidence around the titanium implants nor adverse events were observed; the mean VAS score for satisfaction was 9. All patients enrolled in this trial were esthetically and functionally satisfied with their surgical results, and fixation failure and esthetic dissatisfaction complications were well resolved. Patient-specific titanium showed satisfactory outcomes when used to treat various oral and maxillofacial defects. A 3D printed titanium implant can be effectively used in the reconstruction of the zygoma and mandible instead of autogenous bone without donor site morbidity.

## Introduction

Among the various bones that comprise the skull, the maxilla, mandible, and zygomatic complex are skeletal parts that determine the outline of the facial appearance and enable mastication^1^. When congenital craniofacial deformities occur in the facial skeleton, such as Crouzon or Treacher-Collins syndrome, hemifacial microsomia, or acquired defects due to trauma or tumors, esthetic and functional problems, such as facial disharmony, facial asymmetry, and masticatory problems, can develop^2^.

Autogenous bone grafting or implant placement is the primary method used to treat such defects. Autologous bone grafts are extensively biocompatible; however, there may be problems such as donor-site morbidity, surgical failure, and difficulty in reoperation^3^. In implant placement, there is no donor site morbidity; however, there may be problems in terms of biocompatibility depending on the material and an increase in the surgical cost accompanying the material cost^4^. In addition, both methods are not defect-oriented under conventional surgical methods; hence, residual facial disharmony after surgery cannot be avoided.

Rapid advances in digital technology have led to a paradigm shift in the field of oral and maxillofacial surgery. For example, the spread and development of cone-beam computed tomography (CBCT), computer-assisted design (CAD), computer-assisted manufacturing, and three-dimensional (3D) printers have enabled accurate and rapid surgery^5^. In the case of 3D printers, unlike resin materials that were available in the early days, it is currently possible to print titanium materials that have already been verified for biocompatibility as dental implants^6,7^.

This paper reports the long-term follow-up clinical results of applying patient-specific titanium implants to maxillary, mandibular, and zygomatic defects due to various congenital and acquired causes. The primary outcome variable was the bone fusion between the implant and the bone. Secondary outcomes, search as postoperative infection, satisfaction assessment, osteolysis, subsidence of the titanium implant, and safety, were assessed.

## Results

A total of 16 patients were included (7 women and 9 men) with a mean age of 32.3 ± 14.9 years (range, 9–78 years). Patients were followed up for a mean period of 36.7 ± 20.1 months postoperatively (range, 8–79 months). A screw fracture was found in one of five patients who underwent jaw bone reconstruction during an average observation period of 48 months. Two of the five patients who underwent cheekbone reconstruction underwent revision surgery due to esthetic dissatisfaction during an average observation period of 28 months. There were no side effects for 20 months in six patients in whom excessive bone defects caused by previous plastic surgery were reconstructed with implants.

A total of 28 defect areas were operated on, including five mandibular segments, nine zygomas, ten mandibular bodies, angles, or chins, and four maxillary areas. Regarding the surgical approach, 11 patients underwent reconstruction via intraoral, whereas five, via submandibular approach. The mean surgical operation time was 82 (range, 30–240) minutes. CBCT analysis showed that bone fusion at six months after surgery was 96.5% (27/29). Fourteen patients underwent surgery with 3D-printed implants, and two patients underwent surgery by manufacturing implants using milling methods. Osteolysis was not observed around the 3D-printed titanium implants or milling implants.

The design of the bone-to-implant interface was either a mesh or solid based on whether stability was required. As the rough surface of the mesh-type titanium implant was more likely to osseointegrate into the recipient's bone, we preferred the mesh type on the load-bearing mandible. The electron beam melting (EBM) implants are costlier than the selective laser melting (SLM) implants; however, there was no difference in the clinical results according to the type of implant interface design and the 3D printing method. Commercially available dental implants were installed on 3D-printed titanium implants prior to the surgery. Healing abutments were installed 6 weeks postoperatively without any complications. Customized abutments were made, and the final zirconia implants were fabricated and functioned well. The objective mastication efficiency measured after surgery increased by more than three times compared with that measured before surgery.

The complications of 3D-printed or milling-type titanium reconstruction, such as screw fracture, fixation failure, and postoperative dissatisfaction were experienced by three out of 16 patients. The patient (No. 4) whose left mandible was reconstructed experienced a fracture of the screw that fixed the mandibular implant and the condylar side. However, there were no issues because the implant and bone had already adhered well. The other patient (No. 9) underwent implant removal due to malunion between the zygomatic arch and the titanium implant. In the second surgery, the arch segment and implant were tightened with a wire for fixation. Another patient (No. 10) complained that the right side of the zygoma protruded too much postoperatively. In the second operation, the existing zygomatic implants were removed, grounded well, and fixed again. The patients were satisfied with their facial appearances. The facial appearance and masticatory function remarkably improved, and postoperative recovery was satisfactory in all patients after an average of 27 months. The details of the surgical results and complications are presented in Table 1.

**Table 1:** Surgical results and complications.

	Study population (*n* = 16) Number of Implants (*n* = 28)
**Age at the surgery (years)**	9 to 78 (32.3)
**Sex (M/F)**	9/7
**Surgical approach**	
Intraoral	10
Submandibular	6
**Operating time (minutes)**	30 to 240 (82)
**Hospitalization period**	
2 day	12
3 day	3
4 day	1
**Satisfaction (VAS, 0–10)**	
Functional	8.81 ± 0.83
Esthetic	8.69 ± 1.01
**Bone fusion**	27/28 (96.5%)
**Post-operative infection**	0/28 (0%)
**Implant change**	1/28 (3.5%)
**Re-operation**	3/28 (10.7%)
**Implant Mal-union**	1/28 (3.5%)
**Implant Subsidence**	0/28 (0%)
**Osteolysis**	0/28 (0%)
**Adverse Events**	0/28 (0%)

*VAS* visual analog scale.

## Discussion

This study found that patient-specific titanium implants were successfully used for the reconstruction of craniomaxillofacial defects caused by congenital or acquired dentofacial anomalies during long-term follow-up. Patient outcomes were sustainable in terms of mastication and esthetics.

In this study, the authors made the first attempt to reduce the functional load by placing an implant in a custom titanium body. Two implant shapes were given to the custom titanium body, and temporary crowns were installed after surgery to allow occlusion of the opposing teeth. There were no functional deficits or complications after five years of follow-up. Similarly, in other studies, implant shapes were added to the mandibular molar region, and zirconia implants were shown to function without any problems for one year after restoration^8^. We also attempted to install commercially available dental implants on patient-specific titanium implants without any mismatch between the dental and patient-specific 3D-printed implants. The final implants functioned optimally for at least 30 months.

Most previous studies focused on the calvaria^9,10^, orbital floor^11–13^, maxilla^14,15^, and alveolar bone^16^, where the function load was minimal. There are few reports on custom titanium implants for areas subjected to increased functional loads, such as the zygoma and mandible; however, this study proved that titanium patient-specific implants can function successfully in the zygoma and mandible. It is advantageous in the reconstruction of the mandible, etc., which are subjected to a lot of functional load. As suggested in our previous study, the strength of the patient-specific titanium implant was sufficient to withstand repeated strong loads beyond the masticatory pressure^17^. This is supported by the fact that no mechanical fracture occurred in the single cycle bend test with a force of 4490 ± 198 N and a fatigue test of 1,000,000 cycles with a force of 359 to 1122 N compared to the occlusal pressure (127 to 721 N) of a normal healthy person.

Accurate restoration of the patient's existing appearance is possible. Reconstruction using autologous tissue will determine the result of functional and aesthetic reconstruction according to the surgeon's skill during surgery. However, patient-customized titanium implant using 3D digital technology can be accurately simulated in a virtual environment in advance, so errors are minimized during actual surgery and the appearance of the defect can be accurately reproduced. Previous study of patient specific chin implant revealed accurate as 0.69 mm in mediolateral translation and 2.01° in the yaw orientation^18^. Another study showed that marilla position discrepancy was 1.41 ± 0.58 mm in the patient-specific implant group whereas 2.20 ± 0.94 mm in the conventional group for double-jaw surgery. It also allowed better site adaptation, shorted operating time compared to preformed or pre-bent implants^5,19^.

For autologous bone transplantation such as iliac, tibia, and fibula bone, donor site morbidity is reported to range from 2.5 to 39%^20–23^. In the case of autologous tissue transplantation, there are risks of side effects such as infection of the donor site, delayed healing, decreased sensory and motor functions, and failure to engraft the transplanted tissue^21^. However, in the case of patient-specific titanium implant, donor site morbidity cannot occur at all. It is also supported by previous literature that complications relating to other materials, infection, dislodgment are rare to patient specific implants^24^.

Various adverse events have been reported, including screw fractures, fixation failure, and postoperative dissatisfaction. All complications were resolved by removal of the fixation device or reoperation. The authors explained the following to patients when obtaining informed consent before surgery: (1) Implant failure can lead to infection. (2) We will attempt to fix the implant as precisely as possible using a guide; however, it may not be placed where it was planned. Therefore, reoperation may be necessary. (3) If there is a lesion around the implant, computed tomography (CT) and magnetic resonance imaging may be challenging (artifacts). Therefore, if imaging diagnosis is necessary, the implant must be removed first. (4) Otherwise, osseointegration may not occur. (5) esthetic dissatisfaction can occur. (6) facial asymmetry can also occur. As this was explained in advance, the patients were cooperative.

Titanium, which has a modulus of elasticity similar to that of human bone and significantly high tensile and compressive strengths, has been recognized for its high clinical value as a material in bone reconstruction^25^. The porous structure of titanium is believed to be effective in resolving the mismatch between the modulus of elasticity and human bone^26^. In addition, the mesh structure provided space for bone formation and ingrowth^27^. The porosity of an ideal scaffold for bone regeneration has been reported to be over 66%^28^. In addition, the pore shape of scaffolds influences osteogenesis via cell proliferation. Bidan et al. demonstrated that the optimization of the pore shape improves the growth rate of bone tissue in a porous scaffold, and the cells grow more rapidly within the square pores^29^.

According to our experience, if the entire titanium implant is designed to be porous, friction with the soft tissue occurs during the implant insertion process, which can damage the soft tissue and result in considerable resistance to insertion. Therefore, it is important that the surface in contact with the soft tissue be solid and well-polished. The surface where the titanium implant is in contact with the existing bone can be solid or porous, and each has its own advantages and weaknesses.

In consideration of osseointegration or stress shielding, a porous structure is recommended, but if it is removed owing to patient dissatisfaction and various reasons, it is better to fabricate a solid structure. In the case of mandibular reconstruction with loss of continuity, the bone-contacting surface of the patient-specific implant was porous. Recently, a solid structure has been used for esthetic reasons such as zygomatic bone reconstruction.

In this study, a titanium body was fabricated using SLM and EBM. SLM enables additive manufacturing with metals such as titanium at a high sintering temperature; however, owing to limited dimensional accuracy and poor surface roughness, process improvements are being made to improve its properties^30,31^. EBM uses an electron beam instead of a laser beam to sinter or to fuse the materials. EBM can be used to fabricate complex geometries by scanning each cross-sectional layer selectively, unlike SLM^32^. In this study, the titanium implant did not cause any problems in fixing screw holes and fit. There was no difference in the clinical results according to the 3D printing method.

The limitations of this study were that only prefabricated metals were permitted for temporomandibular total joint implants, and fossa and patient-specific parts were not permitted in countries such as Korea. Although it was possible to connect and apply patient-specific titanium implants to a prefabricated product, legal regulations need to be improved for a safer and more accurate application of patient-specific titanium implants. In addition, the high cost of design and production remains to be resolved^33^. Despite these limitations, our study had several merits. This is the first report to summarize load-bearing 3D-printed titanium implants. Second, this study applied commercially available dental implants to patient-specific 3D-printed titanium comprising a threaded hollow. As revealed in other studies, there is still a need for improvement in volumetric accuracy and screw thread reproducibility; hence, it may not be suitable for use with existing commercially available implants^7^. The restricted biological width/barrier of the mucosal support in contaminated oral environments poses a high risk of infection. In addition, it lacks retrievability when infection or fracture of implants occurs^34^. However, in our study, these volume imperfections were overcome, and the screw of the commercial implant was accurately reproduced and fastened. The patients used implants without any significant functional problems. Our study showed no adverse events in any patient. This retrospective study showed that excellent osseointegration of an implant promotes effective fusion.

Finally, based on the above cases, guidelines for the use of a titanium-based patient-specific titanium implant were presented.

### Indication

1. A continuity defect of the facial bone limited to hard tissue for which reconstruction has already been performed and there is no proper reconstruction option.
2. If there is a mild or moderate bone defect due to previous excessive bone preparation in a patient with facial osteoplasty.
3. In case of high esthetic requirements such as correction of fine skeletal asymmetry.
4. Areas that require functional load bearing, such as the mandible.
5. When simultaneous reconstruction with dental implants is required.

### Contraindication

1. Cases requiring complex tissue reconstruction of hard and soft tissues.
2. Patients with hypersensitivity to titanium material.
3. Patients who require continuous follow-up through radiographic imaging such as CT or MRI (artifact may occur).

## Conclusion

This long-term follow-up study revealed that patient-specific titanium implants have satisfactory outcomes when used for various defects in the oral and maxillofacial regions. A 3D printed titanium implant can be effectively used in the reconstruction of the zygoma and mandible instead of autogenous bone without donor site morbidity.

## Methods

**Figure 1:**
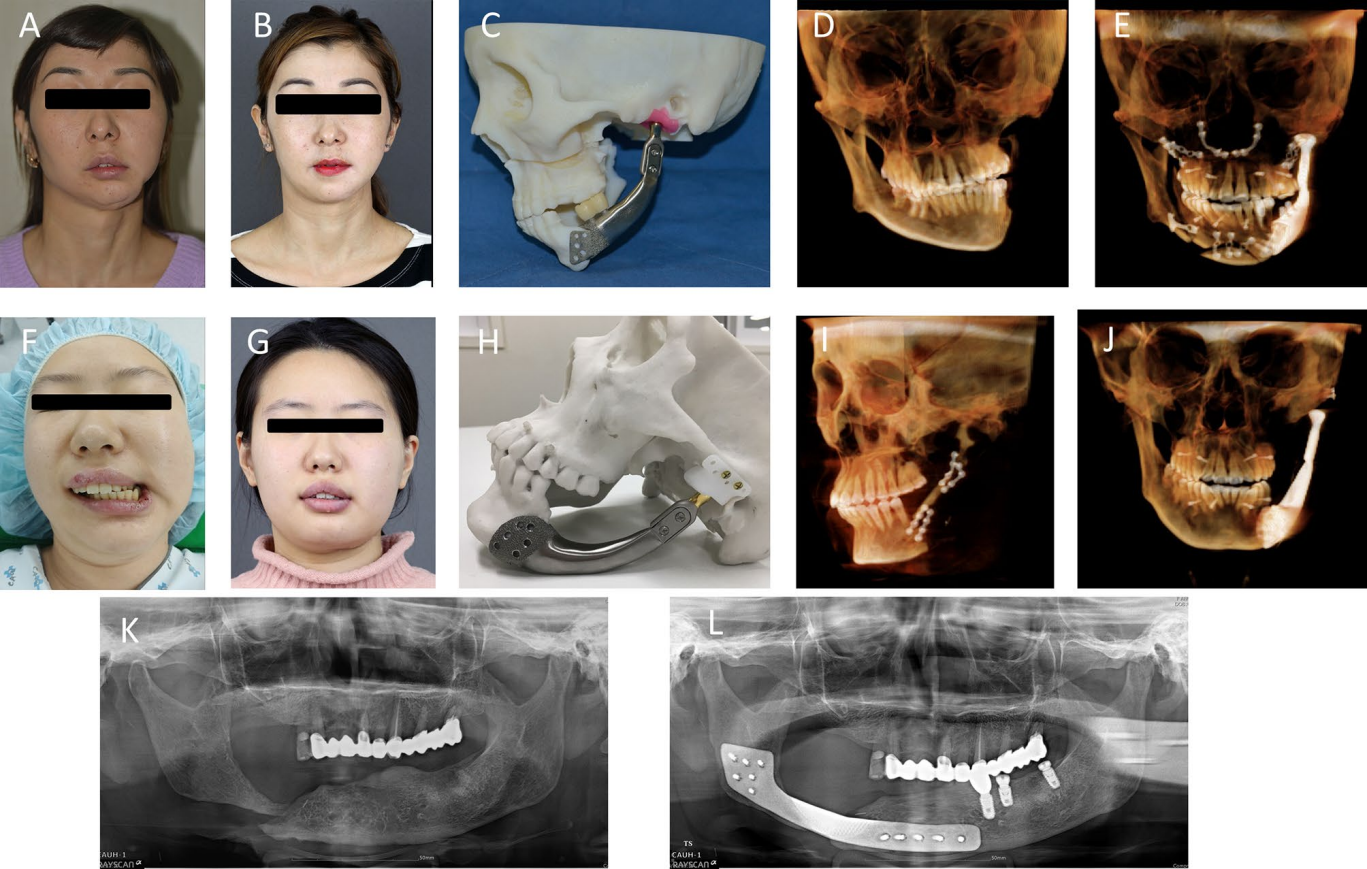
Titanium reconstruction of tumor-induced mandibular defects. (**A**) A 25-year-old female patient was referred for the reconstruction of the left mandible and correction of facial asymmetry; (**B**) Postoperative photograph of the female patient. (**C**) A titanium three-dimensional (3D)-printed mandible was fabricated. The two abutments that faced the central fossa of the maxillary molar were designed for prosthetic restoration. Temporary crowns were also 3D-printed. (**D**, **E**) Preoperative and postoperative 3D reconstructed cone-beam computed tomography (CBCT) images. Reproduced from Lee UL et al. J Oral Maxillofac Surg. 2016 Jul;74(7):1501.e1-1501.e15, with permission of Elsevier Inc. (**F**) A 26-year-old female patient whose mandible was deviated to the left side by more than 10 cm. (**G**) Postoperative facial photograph shows that the face is symmetric, and the mandible is no longer shifted to left. (**H**) To overcome these problems, a titanium 3D-printed mandible was fabricated by Medyssey Inc. The Korean Food and Drug Administration does not approve patient-specific joints as of now. Hence, we connected a prefabricated temporomandibular joint to a 3D-printed mandible. (**I**, **J**) Preoperative and postoperative 3D reconstructed CBCT images. (**K**) A 78-year-old-female patient had a right segmental mandibular defect after a mandibulectomy due to malignancy; (**L**) the mandible was successfully reconstructed with a titanium 3D-printed implant.

This retrospective study was reviewed and approved by Chung-Ang University Hospital’s Institutional Review Board (IRB No. 2103-007-19,358). This case study was conducted using the CARE checklist guidelines. All the methods used in this study were performed in accordance with the Declaration of Helsinki principles. The authors confirmed that written informed consent was obtained from all subjects and/or their legal guardian(s) for information/image publication, and the patients agreed to reveal their facial photos for academic purposes. Sixteen patients (seven men and nine women), who underwent maxillofacial reconstruction with 28 patient-specific titanium implants, were enrolled in this study. Table 2 summarizes the demographic characteristics, defect causes, implant design, combined operations, 3D printing methods, and follow-up periods. CBCT was performed immediately and 6 months after surgery. The fusion between the implant and bone was evaluated using CBCT 6 months after surgery. Implant immobilization, osteolysis, titanium implant subsidence, and safety were also assessed 6 months after surgery. A CT Digital Imaging and Communications in Medicine (DICOM) file, taken 6 months after surgery, was loaded into In Vivo 5 software (Anatomage, San Jose, CA, USA). To assess bone fusion, a multiplanar evaluation at the implant-bone interface was performed. “Fusion” is defined as the case where complete bone contact occurs in > 50% of the total interface area where the implant meets the bone without osteolysis. Osteolysis around the implants and subsidence of surrounding bone were measured using axial, sagittal, and coronal cuts. In reconstruction using a titanium-mandibular body, including a dental implant, the degree of masticatory function was assessed using a chewing ability automatic analyzer (ANA-902, As One, Osaka, Japan). β-carotene gummy jelly, developed exclusively for the test device, was masticated by the patient 30 times and then placed in a beaker with an aqueous solution and loaded onto the test device (Fig. 1). After rinsing and dissolving β-carotene, the device automatically measured the surface area by measuring the β-carotene concentration, which changed according to the photometric voltage. As the jelly’s surface area increased, the red light’s mean voltage decreased. The measured voltage was converted into the surface area of the device using an equivalent ratio. The derived surface area was used as a masticatory function measure.

**Table 2:**
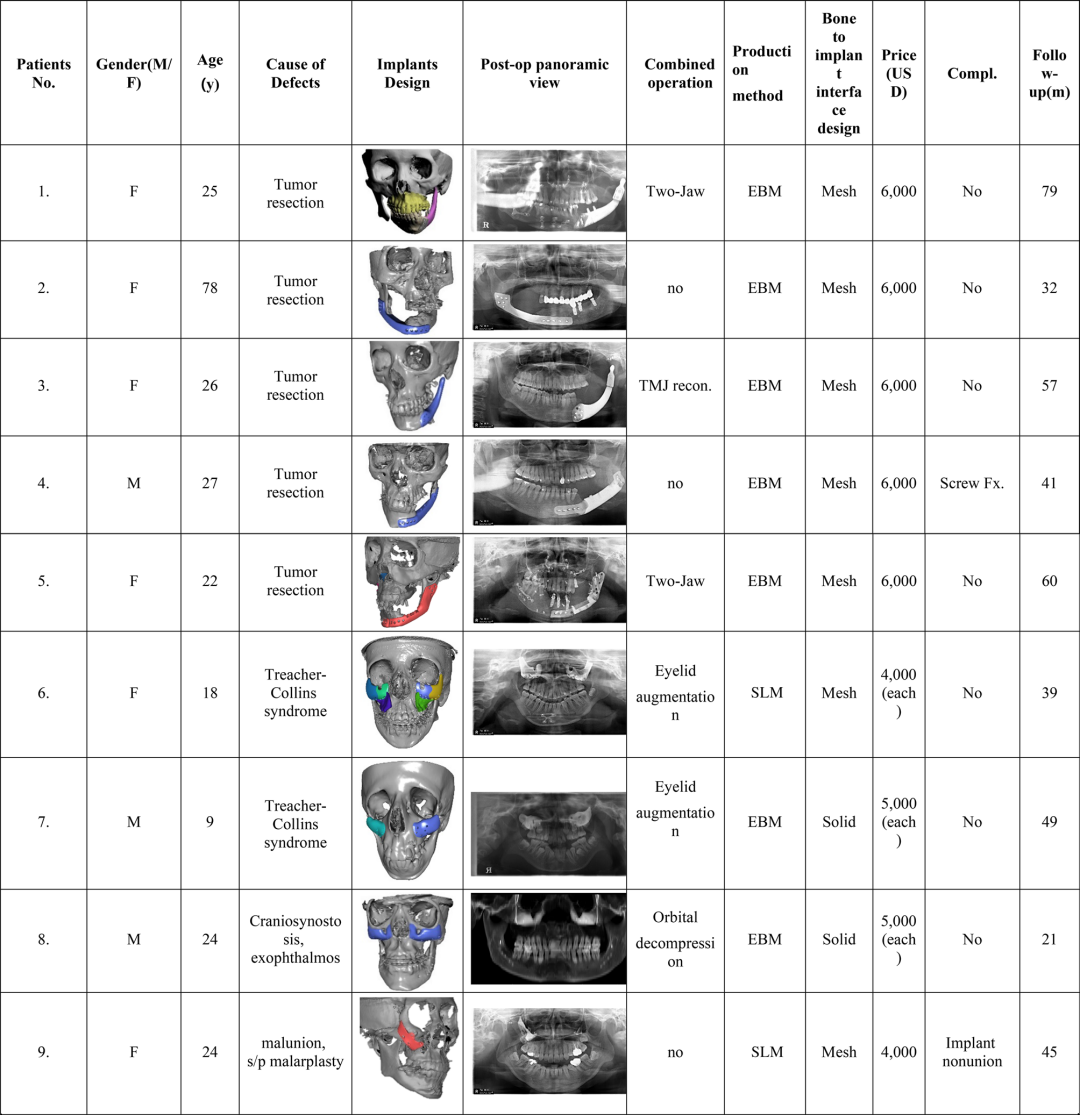

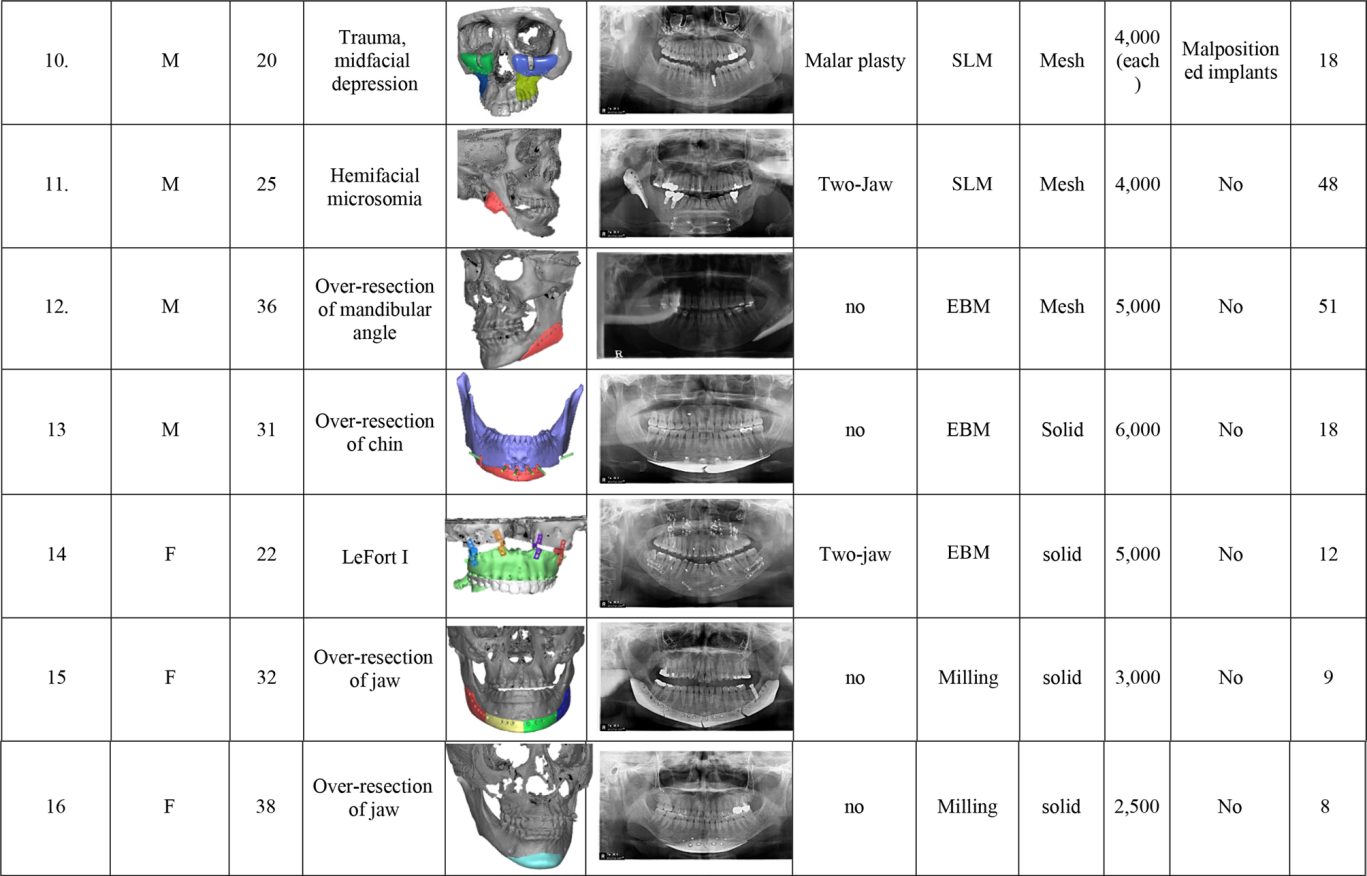
General characteristics of participants.

Abbreviations: *Zyg* zygoma, *Recon* reconstruction, *AVM* arteriovenous malformation, *EBM* electron beam melting, *SLM* selective laser melting.

Using a visual analog scale (0–10), patients were surveyed for the degree of functional and esthetic satisfaction 6 months after surgery.

### Case classification and case presentation

Patients enrolled in the study had maxillofacial defects due to various causes [Crouzon syndrome (1), Treacher-Collins syndrome (2), trauma (1), hemifacial macrosomia (1), malignant tumor resection (2), ameloblastoma (2), arteriovenous malformation (1), iatrogenic facial asymmetry (2), dentofacial deformity s/p plastic surgery (4)]; they are broadly divided into four categories and discussed.

#### Segmental mandibular defect reconstruction

**Figure 2:**
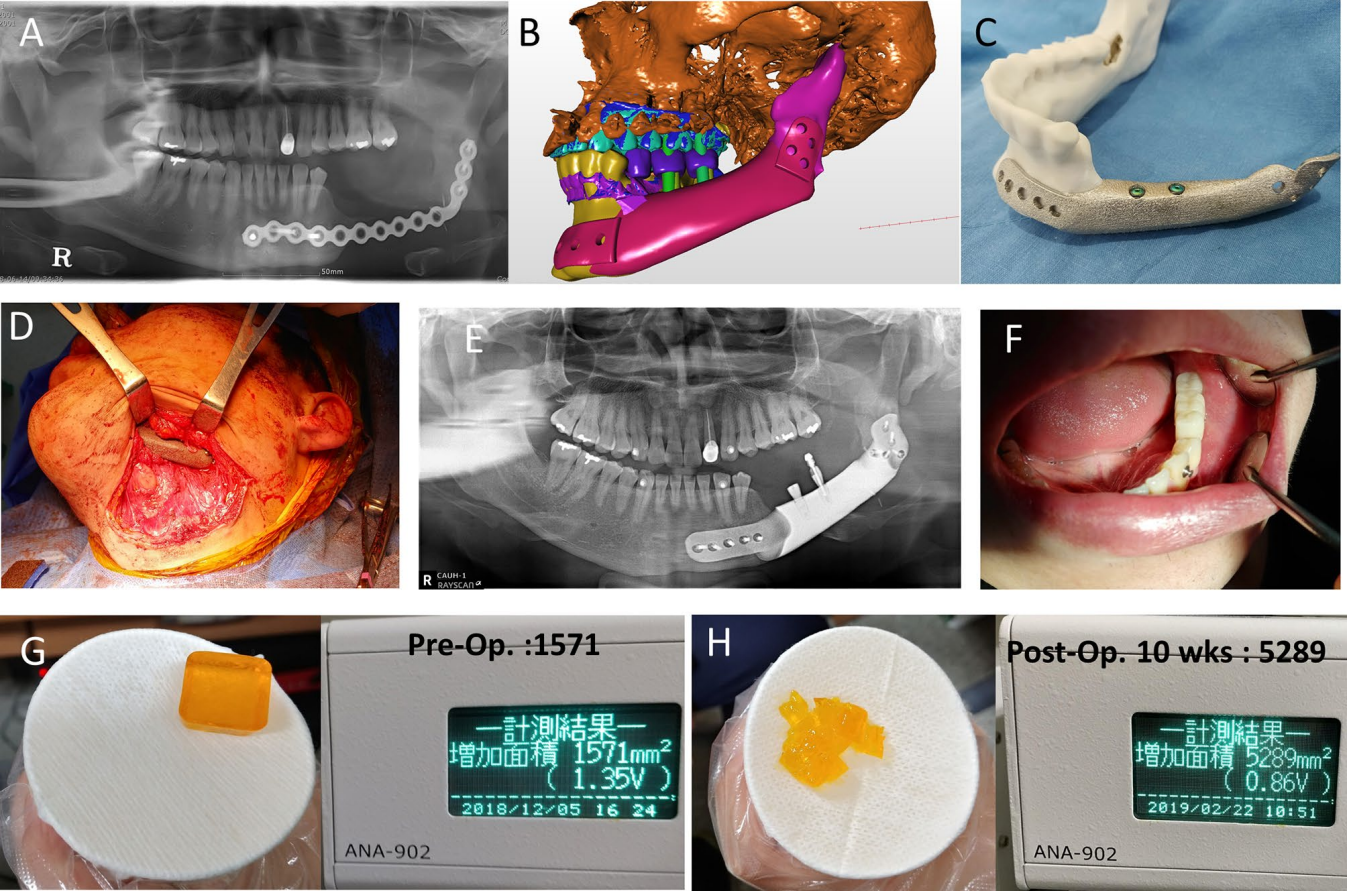
Dental implant on a titanium mandible. (**A**) Initial panoramic radiography of the patient (27/M); s/p ameloblastoma resection and fibula free flap failed. (**B**) To restore the patient’s masticatory function, two holes were designed to place dental implants. (**C**) Two Osstem implants were placed in the titanium mandible; (**D**) A titanium three-dimensional printed mandibular implant with a prefabricated dental implant was placed under general anesthesia. (**E**) The second implant surgery was performed 6 weeks postoperatively. (**F**) Prosthetic treatment was performed using a custom abutment and temporary crowns. (**G**, **H**) The yellow jelly was chewed 30 times by the patient, and the surface area was measured to see how well the jelly was chewed. The mastication efficiency measured after surgery increased three-fold compared with that before surgery.

Five patients had mandibular defects due to tumor resection. Among them, a 25-year-old woman (No. 1) had a defect in the left mandibular body, angle, and condyle resulting from ameloblastoma resection. In addition, the patient had a malocclusion and midline deviation to the side of the defect (Fig. 1A–E). Therefore, orthognathic surgery and mandibular reconstruction were performed with a 3D-printed titanium implant installed with a prefabricated condyle and an artificial abutment that restored the mandibular posterior tooth. Another 26-year-old woman (No. 3) had a case similar to that of case no. 1. However, the left condylar fossa and head were destroyed in her case. As the Korean Food and Drug Administration did not approve patient-specific joints, a custom titanium condyle plus a prefabricated condylar fossa (Zimmer Biomet, Warsaw, Indiana, USA) was used (Fig. 1F–J). A 78-year-old woman (No. 2) had a right segmental mandibular defect after a mandibulectomy due to malignancy. Despite her old age, the mandible was successfully reconstructed using a titanium 3D-printed implant (Fig. 1K,L). A 27-year-old man (No. 4) had mandibular defects due to ameloblastoma resection. Reconstruction was performed using a titanium mandible with a female screw for dental implant placement (Fig. 2). The last patient (No. 5) had a mandibular defect due to arteriovenous malformation. Because mandibular growth was inhibited after mass resection, severe asymmetry and retrognathism were observed. Therefore, orthognathic surgery and titanium mandibular reconstruction were performed.

#### Zygoma reconstruction

**Figure 3:**
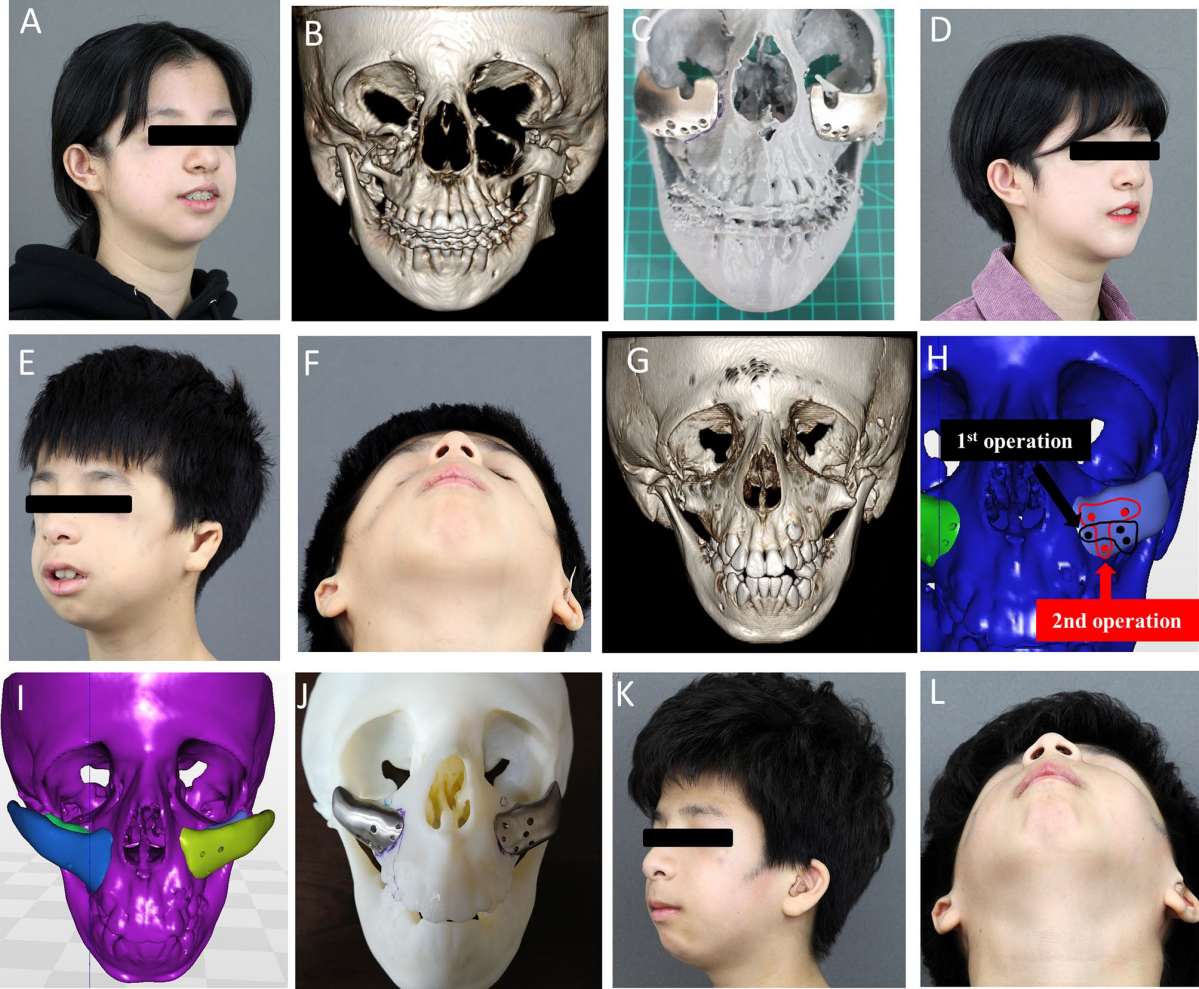
Photographs of patients with Treacher-Collins syndrome. (**A**) An 18-year-old woman with a depressed midface and downward lateral canthus and eyelids. (**B**) A three-dimensional (3D) rendering radiography of the facial skeleton. (**C**) A custom-made 3D-printed zygomatic implant. (**D**) Facial photographs before and 12 months after surgery. The volume of the orbital and zygomatic area increased. The patient has a normal face after simple surgery; (**E**, **F**) Facial appearance of a 9-year-old boy with Treacher-Collins syndrome. (**G**) Underdeveloped zygoma and orbital bone. (**H**, **I**) Considering growth, only three holes out of six were used to fix the zygomatic implant. The other remaining three holes were used for the second surgery. (**J**) A titanium 3D-printed zygoma was fabricated. (**K**, **L**) Facial photographs, taken 3 months after surgery, show that the volume of the zygoma and orbital area increased.

An 18-year-old woman (No. 6) and a 9-year-old boy (No. 7) with Treacher-Collins syndrome were enrolled (Fig. 3A,B). They did not have breathing problems or cleft palate. However, they had severely underdeveloped zygomatic bones, lower orbital rim hypoplasia, brachycephaly, and bitemporal narrowing. In addition, they had downward-angled eyelids, and their upper and lower eyelids drooped. To correct the midfacial deficiency, zygoma and orbital rim augmentation with a 3D-printed titanium implant were performed in patient No. 6 (Fig. 3C,D), who was a woman who completed her skeletal growth. Patient 7 was a 9-year-old boy who also had midfacial deficiency (Fig. 3E–G); therefore, we designed zygoma and orbital rim reconstruction implants to achieve the best results. The implant used in the first surgery was designed so that a new implant could be placed over it if the patient felt that the zygoma volume was insufficient during growth after the first surgery (Fig. 3H). The second implant (Fig. 3I) was virtually designed. There were six holes in the zygomatic implant that were used in the first surgery, three of which were used to fix the zygomatic implant in the first surgery, and the remaining three holes were used in the next surgery (Fig. 3H). The titanium prosthesis was installed and verified in a rapid prototype model (Fig. 3J). The patient’s cheekbone area improved naturally following surgery (Fig. 3K,L).

A 24-year-old man (No. 8) with Crouzon syndrome presented with orbital dystopia, exophthalmos, and malar depression. He had already undergone two-jaw surgery; however, he wanted a more prominent zygomatic buttress and a natural midface. Zygoma augmentation was performed using a zygomatic implant to correct this. Another 24-year-old woman (no. 9) had malunion of the right zygomatic body, which was depressed compared to the opposite side. She underwent bilateral reduction malarplasty at a local clinic. The zygomatic defect was reconstructed with a titanium implant.

A 20-year-old man (No. 10) experienced post-traumatic complications after a traffic accident. The patient underwent open reduction and internal fixation for midface fractures; however, midfacial depression, especially in the zygomatic area, remained as an after effect. Therefore, zygomatic augmentation was performed using a titanium implant.

#### Non-segmental mandibular defect reconstruction

**Figure 4:**
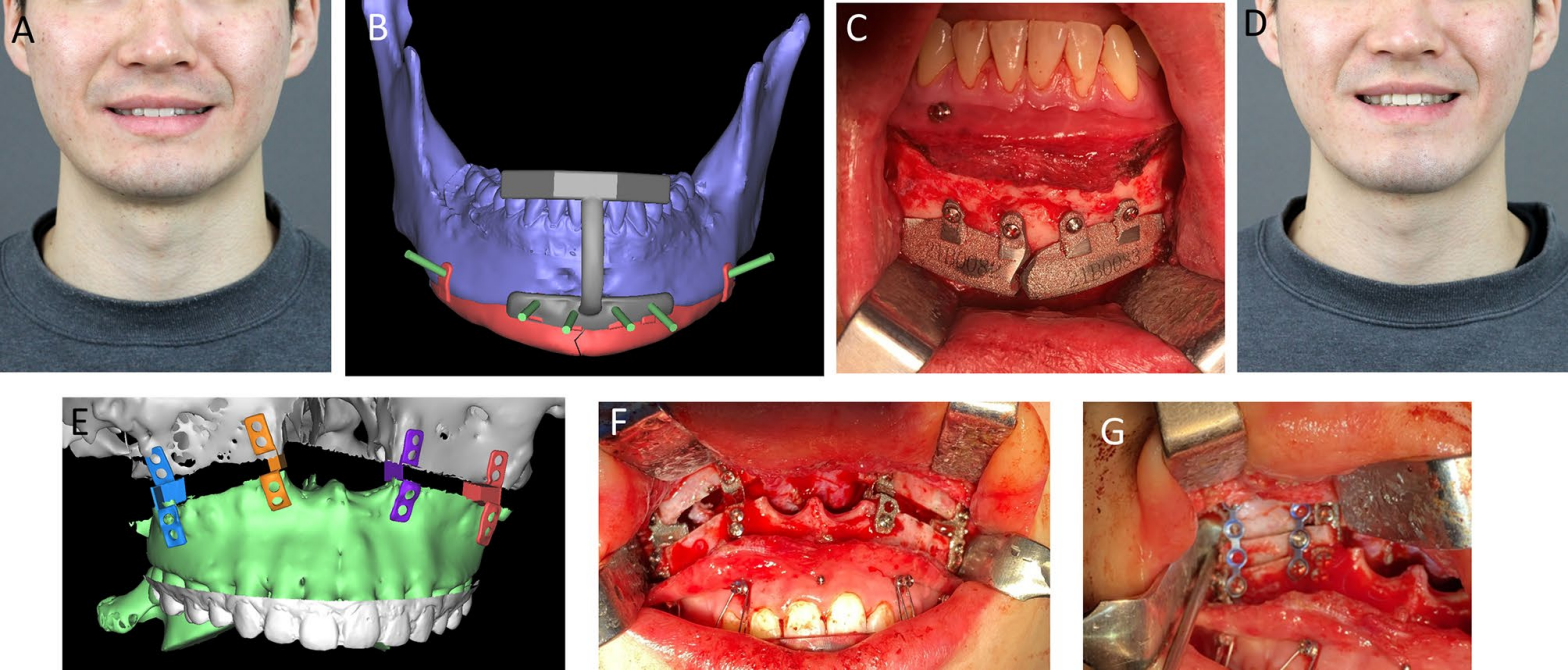
Restoring surgery. (**A**) A 31-year-old male patient underwent genioplasty at a local clinic. He wanted to restore the length of the jaw. (**B**) Computer-aided design of a chin implant and guide. (**C**) Chin augmentation, with two-piece titanium chin implants, was performed. (**D**) Three months after the surgery, the patient was satisfied with the chin length. (**E**, **F**) A 21-year-old woman wanted her upper and lower jaws to be restored the same as before the orthognathic surgery. We planned a downward movement of 5.31 mm at #16 (upper right first molar), 4.69 mm at #26 (upper left first molar), and 3.5 mm at #11 (upper right central incisor) and #21 (upper left central incisor). Three-dimensional (3D)-printed titanium plates that lifted and fixed the maxilla were designed to prevent maxillary collapse and support the block bones. (**G**) The block was well fixed and encased by 3D-printed titanium plates.

A 25-year-old man (No. 11) with hemifacial macrosomia was classified as having Pruzanksky type I mandibular deformity. The patient did not experience any hearing problems. However, the patient had mild hypoplasia of the right ramus and flattening of the condyle and glenoid fossa. The mandible was retrusive, and there was a large discrepancy in the bone volume between the left and right mandibular angles. As the patient had accompanying skeletal malocclusion, unilateral mandibular angle augmentation with a 3D-printed titanium implant was performed by orthognathic surgery. A 36-year-old man (No. 12), who underwent resection of both mandibular angles at a local clinic, had facial asymmetry. The left mandibular angle was over-resected compared to the opposite side. Left-sided angle reconstruction was performed using a titanium implant. Another 31-year-old man underwent a genioplasty at a local clinic as he wanted to restore the jaw length. Chin augmentation with two-piece titanium chin implants was performed (Fig. 4A–D).

#### Downward and counter-clockwise rotational movement of maxilla support

A 21-year-old woman (No. 14) underwent double-jaw surgery at a local clinic and complained that her teeth were not visible, and that her lower jaw was too retrusive after surgery. She wanted her upper and lower jaws to be restored before the surgery. We planned a downward movement of 5.31 mm at #16 (upper right first molar), 4.69 mm at #26 (upper left first molar), and 3.5 mm at #11 and #21 (upper incisors). The 3D-printed titanium plates that lifted and fixed the maxilla were designed to prevent maxillary collapse and support the bone block (Fig. 4E–G).

### Virtual planning

CBCT images (3D eXam, KaVo Dental GmbH, Biberach, Germany) were obtained preoperatively. CBCT data of the maxillofacial regions were obtained with 0.4 mm voxel size and 512 × 512 matrices using 120 kVp (voltage), 11 mA (current), 17.8 s scan time, and a 12-inch detector field. Patient data were stored in DICOM format. Additionally, dentition was scanned using a light emission diode intraoral scanner (I-500, Medit corp, Seoul, Korea). CBCT and scanned data were reconstructed into 3D bone images using the Mimics program (Materialise Co., Leuven, Belgium). After combining the CBCT and occlusion scan data, the dentoskeletal complex was aligned such that the Frankfurt horizontal plane was parallel to the occlusal plane in centric occlusion. Thereafter, a virtual titanium implant was designed to improve asymmetry using the 3-Matics software (Materialise Co, Leuven, Belgium). In the case of artificial abutment or female dental implant, the virtual teeth location and angulation were aligned such that the tooth axis was towards the opposing tooth’s central fossa. The final virtual models were exported as stereolithographic files.

### Fabrication of patient-specific titanium implants

A 3D printing method was adopted using EBM (Odyssey, Seoul, Korea) or SLM (Cusmedi co., Ltd, Gyeonggi-do, Korea). The printing raw material was a Ti-6Al-4 V-ELI medical-grade powder (Arcam A1, Arcam, Molndal, Sweden). In the EBM system, the focused electron beam was raster scanned over each successive layer of powder, which was fed by gravity from powder containers and raked into successive layers of approximately 50 mm thickness. The building components were lowered on the build table with the completion of each successive layer. Each newly raked powder layer was initially raster scanned by the beam after approximately 11 passes at a beam current of approximately 35 mA, to preheat each layer to approximately 60 °C. This preheated layer was usually followed by a beam current of 4 mA. The melt scan was driven by a 3D CAD program that melted only selected areas of the layer to add metal to the build. In the SLM system, the titanium powder for printing was Ti-6Al-4 V alloy powder (M2, Concept Laser, Lichtenfels, Germany). Thin layers of atomized fine titanium powder were evenly distributed on a substrate plate via selective laser melting using a coating mechanism. This takes place inside a chamber containing a tightly controlled atmosphere of inert gas, either argon or nitrogen, at oxygen levels below 500 ppm. Once each layer has been distributed, each two-dimensional slice of the part geometry is fused by selectively melting the powder. This is accomplished with a high-power laser beam, usually a ytterbium fiber laser of hundreds of watts. The laser energy was sufficiently intense to permit the full welding of the particles to form a solid metal. The process is repeated layer-by-layer until the part is complete.

In two patients, implants were fabricated using the milling method (No. 15, 16). These patients wanted reinforcement surgery again after excessive reduction of the lower jaw. In one patient, augmentation was performed only on the chin area; in the other patient, augmentation was performed on the chin, body, and angular areas.

All samples were cleaned in an ultrasonic cleaner with anhydrous ethanol for 15 min. This was because the surface still contained metal particles. Before surgery, the prosthesis was sterilized according to routine procedures in a medical autoclave at 134 °C for more than 3 min.

## References

[1] Ahmad Y, Starbuck JM (2018). Disruption of symmetry: A quantitative assessment of facial skeleton anatomy in children born with unilateral cleft lip and palate. Clin. Anat..

[2] Wedel A, Yontchev E, Carlsson GE, Ow R (1994). Masticatory function in patients with congenital and acquired maxillofacial defects. J. Prosthet. Dent..

[3] Ahmed W, Asim MA, Ehsan A, Abbas Q (2018). Non-vascularized autogenous bone grafts for reconstruction of maxillofacial osseous defects. J. Coll. Phys. Surg. Pak..

[4] Brody HJ (2001). Complications of expanded polytetrafluoroethylene (e-PTFE) facial implant. Dermatol. Surg..

[5] Alasseri N, Alasraj A (2020). Patient-specific implants for maxillofacial defects: challenges and solutions. Maxillofac. Plast. Reconstr. Surg..

[6] Rotaru H, Schumacher R, Kim SG, Dinu C (2015). Selective laser melted titanium implants: a new technique for the reconstruction of extensive zygomatic complex defects. Maxillofac. Plast. Reconstr. Surg..

[7] Park JH (2020). 3D-printed titanium implant with pre-mounted dental implants for mandible reconstruction: a case report. Maxillofac. Plast. Reconstr. Surg..

[8] Rachmiel A, Shilo D, Blanc O, Emodi O (2017). Reconstruction of complex mandibular defects using integrated dental custom-made titanium implants. Br. J. Oral. Maxillofac. Surg..

[9] Park EK (2016). Cranioplasty enhanced by three-dimensional printing: custom-made three-dimensional-printed titanium implants for skull defects. J. Craniofac. Surg..

[10] Huang MT (2019). The potential of the three-dimensional printed titanium mesh implant for cranioplasty surgery applications: Biomechanical behaviors and surface properties. Mater. Sci. Eng. C Mater. Biol. Appl..

[11] Kim YC (2017). The accuracy of patient specific implant prebented with 3D-printed rapid prototype model for orbital wall reconstruction. J. Craniomaxillofac. Surg..

[12] Bachelet JT (2018). Orbital reconstruction by patient-specific implant printed in porous titanium: a retrospective case series of 12 patients. J. Oral Maxillofac. Surg..

[13] Le Clerc N (2020). 3D titanium implant for orbital reconstruction after maxillectomy. J. Plast. Reconstr. Aesthet. Surg..

[14] Gueutier A, Kün-Darbois JD, Laccourreye L, Breheret R (2020). Anatomical and functional rehabilitation after total bilateral maxillectomy using a custom-made bone-anchored titanium prosthesis. Int. J. Oral Maxillofac. Surg..

[15] Fernandes N (2016). Reconstruction of an extensive midfacial defect using additive manufacturing techniques. J. Prosthodont..

[16] Ma J, Ma L, Wang Z, Zhu X, Wang W (2017). The use of 3D-printed titanium mesh tray in treating complex comminuted mandibular fractures: A case report. Medicine (Baltimore).

[17] Lee UL, Kwon JS, Woo SH, Choi YJ (2016). Simultaneous bimaxillary surgery and mandibular reconstruction with a 3-dimensional printed titanium implant fabricated by electron beam melting: A preliminary mechanical testing of the printed mandible. J. Oral Maxillofac. Surg..

[18] Li B, Wang S, Wei H, Zeng F, Wang X (2020). The use of patient-specific implants in genioplasty and its clinical accuracy: a preliminary study. Int. J. Oral Maxillofac. Surg..

[19] Rana M (2015). Increasing the accuracy of orbital reconstruction with selective laser-melted patient-specific implants combined with intraoperative navigation. J. Oral Maxillofac. Surg..

[20] Cricchio G, Lundgren S (2003). Donor site morbidity in two different approaches to anterior iliac crest bone harvesting. Clin. Implant Dent. Relat. Res..

[21] Ling XF, Peng X, Samman N (2013). Donor-site morbidity of free fibula and DCIA flaps. J. Oral Maxillofac. Surg..

[22] Chen YC (2006). Donor site morbidity after harvesting of proximal tibia bone. Head Neck.

[23] Crawford, M. E. in *Lower Extremity Soft Tissue & Cutaneous Plastic Surgery (Second Edition)* (eds G. Dock Dockery & Mary E. Crawford) 225–230 (W.B. Saunders, 2012).

[24] Binder WJ, Kaye A (1994). Reconstruction of posttraumatic and congenital facial deformities with three-dimensional computer-assisted custom-designed implants. Plast. Reconstr. Surg..

[25] Li G (2016). In vitro and in vivo study of additive manufactured porous Ti6Al4V scaffolds for repairing bone defects. Sci. Rep..

[26] Murr LE (2017). Open-cellular metal implant design and fabrication for biomechanical compatibility with bone using electron beam melting. J. Mech. Behav. Biomed. Mater..

[27] Nam JW, Kim MY, Han SJ (2016). Cranial bone regeneration according to different particle sizes and densities of demineralized dentin matrix in the rabbit model. Maxillofac. Plast. Reconstr. Surg..

[28] Kujala S, Ryhänen J, Danilov A, Tuukkanen J (2003). Effect of porosity on the osteointegration and bone ingrowth of a weight-bearing nickel-titanium bone graft substitute. Biomaterials.

[29] Bidan CM (2013). Geometry as a factor for tissue growth: towards shape optimization of tissue engineering scaffolds. Adv. Healthcare Mater..

[30] Calignano F, Manfredi D, Ambrosio EP, Iuliano L, Fino P (2012). Influence of process parameters on surface roughness of aluminum parts produced by DMLS. Int. J. Adv. Manuf. Technol..

[31] Oh JH (2018). Recent advances in the reconstruction of cranio-maxillofacial defects using computer-aided design/computer-aided manufacturing. Maxillofac. Plast. Reconstr. Surg..

[32] Rafi HK, Karthik NV, Gong H, Starr TL, Stucker BE (2013). Microstructures and mechanical properties of Ti6Al4V parts fabricated by selective laser melting and electron beam melting. J. Mater. Eng. Perform..

[33] Tarsitano A, Battaglia S, Sandi A, Marchetti C (2017). Design of a customised bridging mandibular prosthesis for complex reconstruction: a pilot study. Acta. Otorhinolaryngol. Ital..

[34] Goodson AM, Kittur MA, Evans PL, Williams EM (2019). Patient-specific, printed titanium implants for reconstruction of mandibular continuity defects: A systematic review of the evidence. J. Craniomaxillofac. Surg..

